# High incidence of human brucellosis in a rural Pastoralist community in Kenya, 2015

**DOI:** 10.1371/journal.pntd.0009049

**Published:** 2021-02-01

**Authors:** Peninah Munyua, Eric Osoro, Elizabeth Hunsperger, Isaac Ngere, Mathew Muturi, Athman Mwatondo, Doris Marwanga, Philip Ngere, Rebekah Tiller, Clayton O. Onyango, Kariuki Njenga, Marc-Alain Widdowson

**Affiliations:** 1 Division of Global Health Protection, US Centers for Disease Control and Prevention-Kenya, Nairobi, Kenya; 2 Global Health Program, Washington State University, Nairobi, Kenya; 3 Zoonotic Disease Unit, Ministry of Agriculture Livestock and Fisheries, Nairobi, Kenya; 4 Zoonotic Disease Unit, Ministry of Health, Nairobi, Kenya; 5 Center for Global health Research, Kenya Medical Research Institute, Nairobi, Kenya; 6 Department of Health, County Government of Kajiado, Kenya; 7 Bacterial special pathogens Branch, US Centers for Disease Control and Prevention, Atlanta, Georgia, United States of America; University of Iowa, UNITED STATES

## Abstract

**Background:**

Brucellosis occurs globally with highly variable incidence in humans from very low in North America and Western Europe to high in the Middle East and Asia. There are few data in Sub-Saharan Africa. This study estimated the incidence of human brucellosis in a pastoralist community in Kenya.

**Methods:**

Between February 2015 and January 2016, we enrolled persons living in randomly selected households in Kajiado County. Free health care was offered at three facilities in the study area. Those who met the study clinical case definition completed a standardized questionnaire on demographics, clinical history and presentation. A blood sample was collected and tested by Rose Bengal test (RBT), then later tested at the Kenya Medical Research Institute laboratory for *Brucella* IgG and IgM by ELISA. Those who tested positive by both RBT and ELISA (IgG or IgM antibodies) were classified as confirmed while those that only tested positive for IgG or IgM antibodies were classified as probable. Further, sera were tested by polymerase chain reaction using a TaqMan Array Card (TAC) for a panel of pathogens causing AFI including *Brucella spp*. Annual incidence of brucellosis was calculated as the number of confirmed cases in one year/total number in the study population.

**Results:**

We enrolled a cohort of 4746 persons in 804 households. Over half (52.3%) were males and the median age was 18 years (Interquartile range (IQR) 9 months– 32 years). A total of 236 patients were enrolled at three health facilities; 64% were females and the median age was 40.5 years (IQR 28–53 years). Thirty-nine (16.5%) were positive for *Brucella* antibodies by IgG ELISA, 5/236 (2.1%) by IgM ELISA and 4/236 (1.7%) by RBT. Ten percent (22/217) were positive by TAC. We confirmed four (1.7%) brucellosis cases giving an annual incidence of 84/100,000 persons/year (95% CI 82, 87). The incidence did not significantly vary by gender, age and location of residence.

**Conclusion:**

We report a high incidence of brucellosis in humans among members of this pastoralist community. Brucellosis was the most common cause of febrile illness in this community.

## Author summary

Brucellosis is a bacterial disease that affects both humans and animals. Humans get infected via ingestion of unpasteurized animal products from infected animals and direct contact during animal abortions and deliveries. Infected animals shed bacteria for life through milk and during deliveries posing a risk to those with occupational exposure to infected animals. As such, human disease is disproportionately high in regions with high prevalence of animal brucellosis. While human brucellosis is distributed globally, incidence is low in North America and Western Europe and high in Asia and the Middle East where the disease is endemic. Data from Africa are scarce. We set out to estimate the incidence of brucellosis in a pastoralist community with documented high *Brucella* sero-prevalence in humans and livestock. We followed up a cohort of 804 households for one year and tested household members who became ill in three designated health facilities. We estimated an incidence of 84 cases per 100,000 persons per year in this community. We also found that *Brucella* was the most common pathogen among persons who had febrile illness highlighting the importance of this pathogen in this rural pastoralist community. No brucellosis intervention measures were being implemented.

## Introduction

Brucellosis is a common bacterial zoonosis caused by multiple *Brucella* spp, endemic in domestic and wild animals where it causes abortions, reduced fertility, poor weight gain and reduced milk production resulting in substantial productivity and economic losses [1]. Human transmission occurs via ingestion of unpasteurized animal products and direct contact during animal abortions and deliveries. Human infection is characterized by an acute or chronic debilitating illness characterized by fever, joint pains, night sweats, fatigue, headache, and weight loss persisting for weeks to months [2].

While human brucellosis is distributed globally, incidence is variable across regions [3,4]. Brucellosis incidence or rates ranges from low in North America and Western Europe (≤ 0.1 cases per 100,000 population), moderate in Central and Southern Latin America and parts of South Eastern Europe (3.5–35 cases per 100,000 population) and endemic in Asia and the Middle East where some of the highest estimates (>250 cases per 100,000 population) have been reported [4–7].

There are scarce data on incidence of human brucellosis in Africa, with typically only subnational data available from a few countries. Variable annual human brucellosis incidence has been reported, ranging from 3.5 per 100,000 population in Tunisia to 8.4 per 100,000 population in Algeria and 35 per 100,000 population in Chad and Tanzania [3,4,8]. Notably, most studies in Sub-Saharan Africa focused on *Brucella* antibody sero-prevalence in humans and livestock, data that provide an insight into the inferred high burden of brucellosis particularly among rural populations who heavily rely on livestock for their livelihood [9–11].

Data on incidence of human brucellosis in Kenya are not available. However, *Brucella* antibody sero-prevalence data suggest widespread exposure to *Brucella* spp in human and animal populations in Kenya. A recent review of occurrence of human brucellosis reported low sero-prevalence (<1%) in Western Kenya and Nairobi, to high sero-prevalence (up to 46%) in most regions under nomadic pastoralism in Kenya [11]. High *Brucella* sero-prevalence was reported in camels (10–38%), cattle (3–15%) and goats (4–17%) raised under pastoral and agro-pastoral systems and low seroprevalence reported in cattle (<1–9%) and goats (<2%) raised in small holder intensive farms [11–14]. These data on sero-prevalence taken together with the intricate relationships pastoralists have with their livestock and cultural practices around consumption of dairy products suggest high transmission rates in pastoralist communities [10,12,13,15].

The aim of this study was to estimate the incidence of and risk factors for brucellosis in a pastoralist community with documented high *Brucella* sero-prevalence in humans and livestock.

## Methods

### Ethics statement

The study received ethical approval by the Kenya Medical Research Institute Scientific Ethical Review Committee and Centers for Disease Control and Prevention Institutional Review Board. Approval was also obtained from the Kenya Ministry of Health, the Ministry of Agriculture Livestock and Fisheries and the County Government of Kajiado. Written informed consent was obtained from all enrolled participants.

#### Study site

Between February 2015 and January 2016, we established a prospective community cohort at Mashuru Sub-County of Kajiado County, with clinical follow up of participants at study health facilities. An earlier study in the county reported *Brucella* seroprevalence of 15% in humans and 3% in livestock (cattle, sheep and goats); >50% of households in areas surrounding Mashuru sub-county had at least one sero-positive animal [12].

The sub-county covers 2903km^2^ with an estimated total human population of 50,245 (density of 17.3/km^2^) based on the Kenya 2009 census. Most parts of Mashuru sub-county, which is inhabited by nomadic pastoralists, have semi-arid conditions and receive an average of 400–500 mm rainfall per annum. Livestock-keeping and subsistence crop farming are the main agricultural activities.

#### Selection of study locations and households

**Fig 1:**
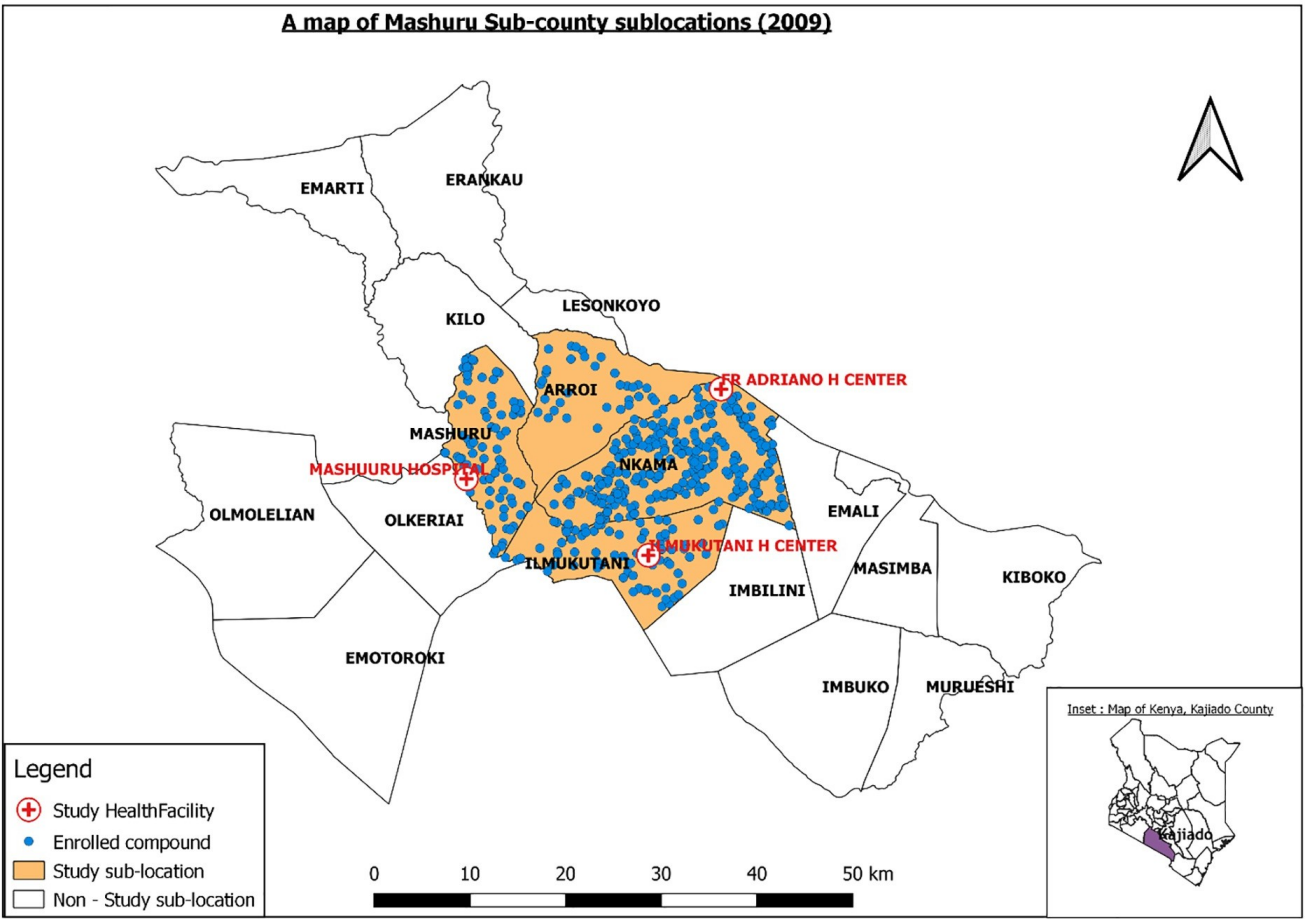
Map of Mashuru sub-county showing the study sublocations and enrolled households. Inset is a map of Kenya showing Kajiado county.

Four of 17 sub-locations (Arroi, Ilmukutani, Mashuru and Nkama), with a total human population of 15,036 living in 3210 households were selected in consideration of existing health centers. Total human population was 1321 in Arroi, 2266 in Ilmuktani, 2722 in Mashuru and 8727 in Nkama [16]. A sublocation is the smallest administrative unit in Kenya. We registered 500 compounds for longitudinal follow up (Fig 1). A compound comprised of a cluster of households of relatives or clanmates living in close proximity in an enclosure and who most often pooled and reared their livestock into one herd. The number of compounds targeted in each sub-location was weighted by population size based on the 2009 Kenya population census data [16]. In order to identify compounds to register, we generated 500 random geographical coordinates using ArcGIS (ESRI, Redlands, CA, USA) corresponding to number of compounds for each sublocation including 37 compounds in Arroi, 74 in Ilmuktani, 74 in Mashuru and 315 in Nkama sub-location. For each coordinate we selected the closest compound for enrollment. All households in a selected compound were invited to participate in the study. Each consenting household was assigned a unique household identifier.

#### Recruitment of human cohort

All household members in a recruited household were eligible for enrollment into the study. Each household member was assigned a personal identification number and issued a card bearing their personal details for use during the health center visits. A baseline questionnaire was administered to the head of each household to collect demographic and health seeking behavior data on each household member and animal ownership data. Households were sensitized on clinical symptoms associated with brucellosis and asked to present at the only three health facilities distributed across the study area for care if they became ill with signs or symptoms compatible with brucellosis.

#### Longitudinal follow up in humans

A register of all household members was stored in all three study health facilities for use if any enrolled person presented for care. A study nurse and a laboratory technologist were placed in each health center to carry out study procedures. Enrollment into the study was performed Monday through Friday each week. A public health officer contacted all the household heads every two weeks to enquire of any illness and remind the household members to visit the study health facility for any illnesses.

#### Clinical case definition for enrollment at the health centers

For eligibility for enrollment, we used a non-specific clinical case definition that included a member of a registered household aged ≥ 1 year presenting at any of three study health facilities with temperature > 38°C at the time of clinic visit, or history of recurrent or continuous fever, and no identified cause of fever such as diarrhea and respiratory illness, and any two of: night sweats, joint pains, joint swelling, headache, fatigue, anorexia, muscle pain, or back pain.

#### Specimen and data collection

We enrolled into the study those who met the case definition and consented. We collected demographic data, history of illness and clinical presentation and contact with animals and animal products via a questionnaire on smartphones. A blood specimen was collected and centrifuged for serum separation at the health facility.

### Diagnostic investigations

Initial serum testing was conducted at the health facility and two milliliters of serum were stored at -20°C at the health center and later shipped for storage and testing at the Center for Global Health Research, Kenya Medical Research Institute (KEMRI) laboratory in Kisumu.

#### Testing at health facility for case management

An aliquot of each serum sample was tested for brucellosis by Rose Bengal Plate Test (RBT) [VLA, UK] agglutination assay [17] and for malaria by a rapid diagnostic test (RDT) [Carestart] at the health facility to facilitate case management.

### Testing at CGHR KEMRI laboratories in Kisumu

#### Brucella ELISA

All specimens were tested using IBL-America IgG and IgM enzyme-linked immunosorbent assay (ELISA) kits according to manufacturer’s recommendation as previously described [12].

### Multi-pathogen testing by TaqMan array cards for Acute Febrile Illness (AFI TAC)

Total nucleic acid was extracted from 166 μl of sera in a KingFisher ML extraction platform (Thermo Scientific, Waltham, MA) using a MagMAX nucleic acid isolation kit (Life Technologies, Carlsbad, CA). Briefly, 166 μl of sample was mixed with 433 μl of lysis-binding solution and was then washed once with 600 μl wash solution 1 and twice with 450 μl wash solution two and was eventually eluted in 200 μl elution buffer. Molecular testing was performed by polymerase chain reaction using acute febrile illness TaqMan Array Card (AFI TAC) diagnostic V2 as described previously [18]. The TAC cards used in this study detects 17 viruses, 8 bacteria and 3 protozoa (S1 Fig). We assessed detection of *Brucella* DNA and other etiologies of febrile illness among all enrolled participants [19–21].

### Brucellosis case classification

We used a modified case definition to the World Health Organization for confirmed cases [22]. We defined confirmed cases as those who met the study clinical case definition and had confirmatory laboratory diagnosis by either (i) testing positive for anti-*Brucella* agglutinating antibodies by RBT and anti-*Brucella* IgG or IgM antibodies by ELISA. We defined probable cases as those who met the study clinical case definition and were sero-positive for anti-*Brucella* IgG antibodies by ELISA only, or IgM only but negative by RBT.

### Data analysis

All analyses were done using STATA 12 (Stata Corporation, College Station, TX, USA). Descriptive statistics were conducted for socio-demographic and other characteristics for the study cohort and those enrolled with febrile illness. Cases were categorized as confirmed, probable, indeterminate and negative for brucellosis. Confirmed *Brucella* positivity was determined as the proportion of confirmed cases against all persons tested.

#### Calculating annual incidence of brucellosis in humans

Annual incidence was estimated as the number of brucellosis confirmed cases in the study year (February 2015 –January 2016) /total number at risk in the study population per 100,000 population. The total number at risk in the population was obtained from the baseline survey where all members in the registered households were listed, provided with a personal identification number and were eligible for enrollment into the study if they met the case definition for the brucellosis upon evaluation by the study nurse. We calculated the incidence by gender, age (categorized as <20, 21–40, 41–60 and >60 years) and location of residence and reported with 95% confidence interval estimates. The estimated incidences were compared, and incidence ratios were calculated across the gender, age categories and location of residence and 95% CI reported.

## Results

### Study cohort demographic information

In February 2015, a cohort of 504 compounds comprising a total of 804 households in the four locations were registered for follow up. The total number of household members was 4,746; 52.3% were males and the median age was 18 years (upper and lower bounds of 25^th^ and 75^th^ percentile 9 months, 32 years) (Table 1). The median number of persons in each household was 6 (25^th^ and 75^th^ percentile 1–19). At enrollment, majority of households 780 (97%) owned at least one type of livestock with 95% (n = 764), 84% (n = 674) and 81% (n = 654) owning goats, cattle, and sheep, respectively.

**Table 1:** Demographic characteristics and household livestock ownership of the study cohort and enrolled patients, Kajiado 2015–2016.

Demographic characteristics	Cohort n (%) N = 4746 N =	Suspected brucellosis cases N = 236
Age (median, IQR) Years	18 (0.8, 32)	40.5 (28, 53)
**Gender**		
Male	2,484 (52.3)	85 (36.0)
Female	2,262 (47.7)	151 (64.0)
**Education level completed**		
No formal education	2,642 (55.7)	124 (52.5)
Primary	1,262 (26.6)	72 (30.5)
Secondary	5,49 (11.6)	31 (13.1)
College	293 (6.2)	9 (3.8)
**Employment status**		
Working on farm	1527 (32.2)	109 (46.2)
Non skilled	185 (3.9)	61 (25.8)
Skilled	442 (9.3)	31 (13.1)
Students and minors	2591 (54.6)	35 (14.8)
**Total household population by Livestock ownership**		
Own any livestock	4626 (97.5)%)	154 (66.1)
Own goats	4533 (95.5)	151 (64.8))
Own cattle	3989 (84.0)	142 (60.9)
Own sheep	3897 (82.1)	133 (57.1)

IQR–Inter quartile range

### Enrolled patients’ demographic information

Between February 2015 and January 2016, 236 persons from 178 (22%) registered households presented at one of three health care facilities and met the study clinical case definition and were enrolled. Of these, 64% were females and the median age was 40.5 years (25^th^ and 75^th^ percentile 28, 53 years) (Table 1). Over half of the participants (52%) had no formal education and 46% reported working full time on the farm (Table 1).

Among patient households, 66% owned at least one livestock type with 65%, 61% and 57% of the participants owning goats, cattle, and sheep, respectively, in their households in the three months prior to the health facility visit (Table 1). About 40% were referred to the health center by the study personnel during routine household visits. Almost all (99%) participants reported consuming animal milk, with 78% reporting drinking cow milk more than three times a week. Over 91% reported consuming only boiled cow milk. Over half of the participants reported abortions among their livestock in the previous year; 23% reported abortions in cattle, 42% in goats, and 23% in sheep.

### Clinical presentation of suspected brucellosis cases

**Fig 2:**
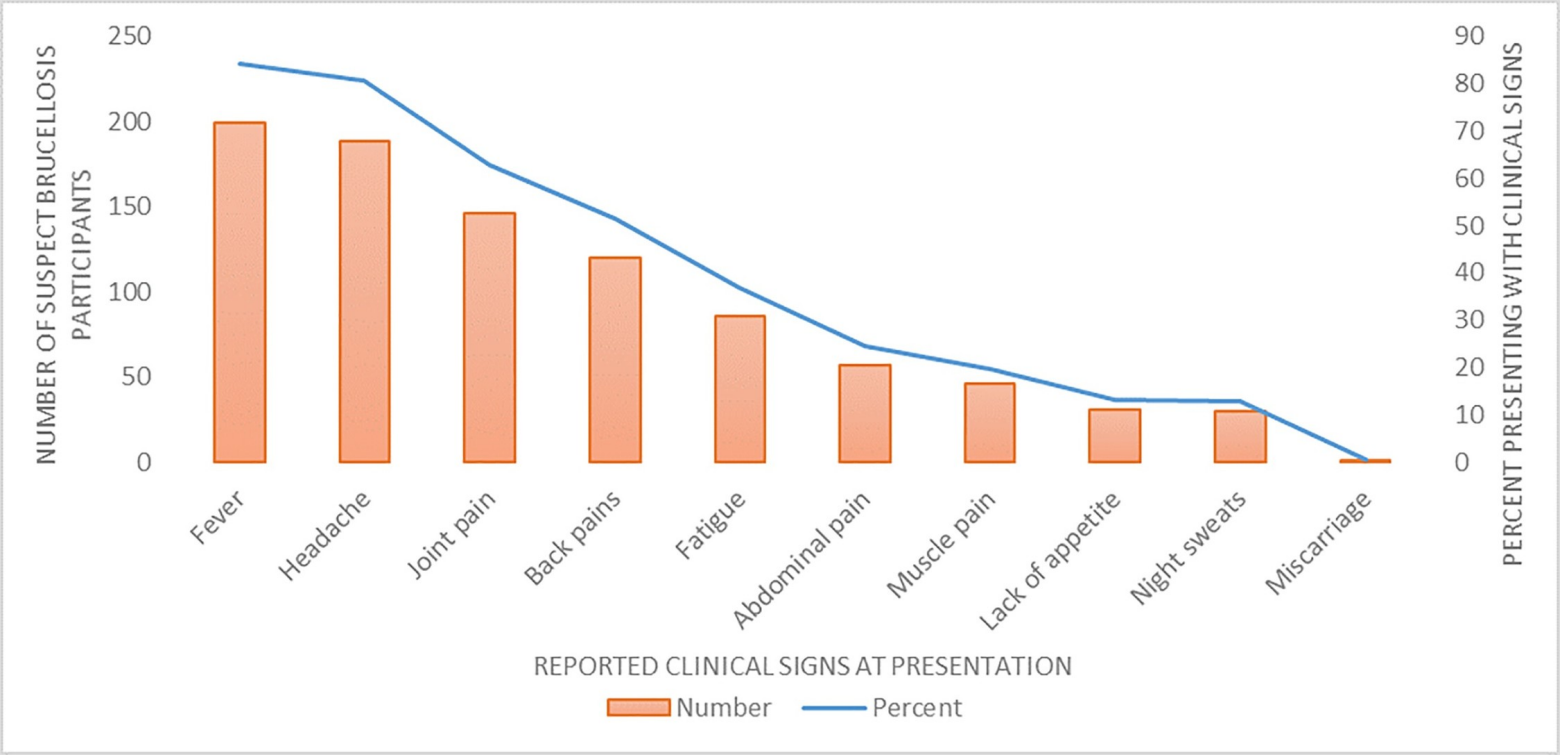
Reported clinical signs of enrolled cases at presentation at health facilities (N = 236).

At the time of visiting the health facility, the most frequently reported clinical signs/symptoms among the 236 enrolled were: any fever in 199 (84%), headache in 188 (81%), joint pain in 146 (63%) and back pain in 120 (51%) [Fig 2]. The median time of presentation to the health facility from date of onset of symptoms was 7 days (25^th^ = 4 and 75^th^ percentile = 14 days), and this did not differ by sex or age (p = 0.05). In total, 102 (44%) of the patients reported having experienced similar symptoms in the last 12 months, with over half (59%) having experienced the symptoms two or more times. Most (89%) had previously sought medical care with only 36% reporting symptoms of previous illness having resolved.

### *Brucella* test results

*Brucella* serology: Of the 236 cases enrolled, 39 (16.5%) tested positive by *Brucell*a IgG ELISA, five (2.1%) by IgM ELISA and four (1.7%) by RBT (Table 2). Four of 236 participants (1.7%) were classified as confirmed cases and 39 (16.5%) classified as probable cases.

**Table 2:** Distribution of laboratory test results by brucellosis test assay for febrile cases, Kajiado 2015–2016 (n = 236).

Brucellosis Case classification	Laboratory test assay			
	Rose Bengal Test (RBT) N = 233	*Brucella* IgM ELISA N = 236	*Brucella* IgG ELISA N = 236	Total Number by brucellosis case classification n(%)
Confirmed^ᵦ^	2	2	0	2
	1	1	1	1
	1	0	1	1
**Total Confirmed**	**4**	**3**	**2**	**4 (1.7)**
Probable^†^	0	0	37	37
	0	2	0	2
**Total Probable**	**0**	**2**	**37**	**39 (16.5)**
**Total Negative**	**229**	**231**	**197**	**193 (81.8)**
**Overall Total no. positive by diagnostic assay (%)**	**4 (1.7)**	**5 (2.1)**	**39 (16.5)**	**-**

^ᵦ^ **Confirmed** cases tested positive by RBT and IgG or RBT and IgM ELISA.

^**†**^ **probable** cases tested positive by ELISA (IgG) only, or IgM only but negative by RBT.

#### TAC testing for *Brucella spp*

AFI TAC was performed on 217 (91.9%) sera with sufficient volume and *Brucella* spp DNA was detected in 22 (10.6%) of the samples. The distribution of *Brucella spp* DNA detected by case classification is shown in S1 Table.

#### Test results for other etiologies of febrile illnesses

Fourteen (6%) of 233 febrile cases tested positive for malaria with the RDT and plasmodium DNA was detected in two of 217 sera tested by TAC for a total of 16/236 (7%) enrolled cases testing positive for malaria. From the TAC testing, six specimens had HIV-1 virus detected (Table 3). Of these, five had HIV virus alone detected and two were co-detection of *Brucella* spp and HIV virus. Two samples had two pathogens detected; *Brucella* spp and *Plasmodium* spp (n = 1), and *Rickettsia* spp DNA and hepatitis E virus (n = 1). There were single detections each of Rift Valley fever virus, *Yersinia pestis*, and *Salmonella* spp (Table 3). No pathogen was detected by TAC in the majority of cases (85%).

**Table 3:** Summary of etiologies of fever among patients by Acute Febrile Illness TacMann Array card (AFI TAC) testing in Kajiado, Kenya, 2015–2016 (n = 217)*.

Pathogen(s) detected	AFI TAC test results n (%)
*Brucella spp*	20 (9.2)
*Plasmodium spp*	1 (0.5)
*Plasmodium spp* and *Brucella spp*	1 (0.5)
HIV-1	5 (2.3)
HIV-1 & *Brucella spp*	1 (0.5)
*Rickettsia spp* & Hepatitis E	1 (0.5)
West Nile Virus	1 (0.5)
Rift Valley Fever virus	1 (0.5)
*Salmonella spp*	1 (0.5)
*Yersinia pestis*	1 (0.5)
Negative	184 (84.8)

*19 cases had insufficient sera for TAC testing.

### Demographic and clinical characteristics of confirmed brucellosis cases

The median age of the four patients with confirmed brucellosis was 34 years (25^th^ Percentile = 20 years and 75^th^ percentile = 41.5 years) and 3 (75%) were females. Three of the confirmed cases reported not owning any livestock at household level. The median days of presentation to the health facility since symptom onset was 9 days (25^th^ = 4 days and 75^th^ = 21 days). All the patients presented with fever and headache. Other common presenting signs were joint pains, fatigue, night sweats and back pain (Table 4).

**Table 4:** Demographic, clinical characteristics and laboratory assessments of confirmed brucellosis cases in Kajiado, Kenya 2015–2016 (n = 4).

					Laboratory assessments		
Patient # age[y]/sex	Occupation	Days since onset of symptoms	symptoms	RBT	ELISA IgM	ELISA IgG	TAC
							Brucella spp*
1. 10/M	Child	4	Fever, Headache, Joint pains	+	-	+	NT
2. 38/F	Housewife	4	Fever, Headache, Joint pains, night sweats, lack of appetite, abdominal pains, fatigue	+	+	-	-
3. 45/F	Housewife	14	Fever, Headache, Muscle pain, fatigue, back pains	+	+	-	+
4. 30/F	Housewife	28	Fever, headache, joint pains, lack of appetite, back pains	+	+	+	+

### Demographic and clinical characteristics of probable brucellosis cases

The median age among the 39 probable cases was 50 years (25^th^ = 35 and 75^th^ percentile = 64) and 23 (59.0%) were females. The median days of presentation of probable cases to the health facility since symptoms onset was 7 days (25^th^ = 3 and 75^th^ percentile = 14). Seventy-seven percent presented with any fever, 79% with headache, 59% with joint pain, 59% with back pain, and 25.6% with muscle pain among others (S2 Table). Forty six percent (n = 18) of probable cases reported having had a similar illness in the previous 12 months with 12/18 (67%) reporting two or more episodes of similar illness. In 7/18 (39%) probable cases, illness symptoms had resolved.

### Incidence of human brucellosis

The annual incidence of brucellosis in this population was estimated to be 84/100,000 persons/year (95% CI 82, 87). The annual incidence by gender, age and location is shown in **Table 5**. There were no confirmed cases among those living in the Arroi location (Fig 1). The risk of brucellosis did not vary significantly by age, gender or location of residence.

**Table 5:** Annual Incidence of brucellosis in humans by gender, age and location in Kajiado Kenya, 2015–2016.

Characteristic	Population in the study cohort	Total enrolled febrile illness cases n (%) N = 236	Number of confirmed Brucellosis cases N (%) (N = 7)	Point estimate annual incidence per 100,000 [95% CI)	Incidence ratio Estimate (95% CI)
Gender					
Male	2484 (52.3)	85 (36.0)	1 (1.2)	40 [38,43]	Ref
Female	2262 (47.7)	151 (64.0)	3 (2.0)	133 [128,138]	3.3 [0.2–173.0]
Age group					
≤20	2542 (53.6)	30 (12.7)	1 (0.04)	39 [37,42]	Ref
21–40	1388 (29.2)	83 (35.2)	2 (4.8)	144 [138,151]	3.6 [0.19–216.1]
41–60	570 (12.0)	79 (33.5)	1 (1.3)	175 [165,187]	4.4 [0.05–350.1]
>60	246 (5.2)	44 (18.6)	0 (0.0)	0	-
Location					
Arroi	425 (8.9)	9 (3.8)	0 (0.0)	0	-
Ilmuktani Mashuru Nka	831 (17.5)	93 (39.4)	1 (1.1)	120 [17,853]	3.2 [0.04–255.3]
Mashuru	787 (16.6)	54 (22.9)	2 (3.7)	254 [64,1014]	6.8 [0.35–405.2]
Nkama	2703 (56.9)	80 (33.9)	1 (1.2)	37 [5,262]	Ref

## Discussion

We report a high annual incidence of human brucellosis of 84 per 100,000 persons in a pastoralist community in Kenya for which limited data had previously been available. Using a multi-pathogen molecular assay designed for detecting 26 pathogens associated with acute febrile illness, *Brucella* spp was the most common pathogen (detected in 10.1% of samples), highlighting its importance as a leading cause of febrile illness in this community. Other pathogens detected by TAC included *Plasmodium* (0.9%), HIV-1 (2.8%) and different combinations of co-detections of *Brucella* spp and *Plasmodium*. Of note, the majority (77%, n = 17) of those who had *Brucella* spp DNA detected did not have detectable antibodies by either serological assay. [23,24].

Diagnosis of brucellosis is complex and confirmatory diagnosis is made by *Brucella* culture or achieved through a series of serological tests. For these patients in a high sero-prevalence area who presented with a clinical syndrome compatible with brucellosis, we applied specific laboratory confirmation criteria for confirmed cases that included both detection of agglutinating and non-agglutinating antibodies. Based on these criteria, 16% of cases who were seropositive by IgG or IgM ELISA only were classified as probable cases. Non-agglutinating IgG antibodies are associated with prior exposure to *Brucella*, incomplete treatment or chronic brucellosis with focal complications [20,25]. We hypothesize that a proportion of these probable cases likely represent chronic brucellosis, relapsing brucellosis and/or occupational exposures in this endemic area in persons who may ultimately require brucellosis treatment. Hence, the incidence reported here (derived from confirmed cases only) may underestimate the true incidence in this community by more than threefold.

The reported incidence in this pastoralist area was 2.5 times higher than 33/100,000 population reported in 2008 and 35/100,000 persons in 2014 in the Kilimanjaro region in the neighboring country of Tanzania [8]. Variable incidence across regions and within a country is commonly demonstrated [4]. For example, Egypt reported human brucellosis incidence of 0.3–70/ 100,000 persons, Iraq 52–267/100,000 persons, Saudi Arabia 6–149/100,000 persons and Greece 4–32/100,000 persons variable by sub-national regions [4]. The higher rates are often reported among nomadic pastoralists and lower rates among urban populations with limited contact with infected livestock and unpasteurized dairy products [5,10,12,13,26,27]. Generally, the occurrence of human brucellosis is highly correlated with *Brucella* sero-prevalence in livestock, where infected animals are constantly shedding bacteria in milk and at parturition increasing the likelihood of infection among humans. However, the majority of participants (97.5%) in this pastoralist community owned at least one livestock type at household level; this limited the power to estimate incidence by livestock owned. A 2013 study conducted in three counties in Kenya, including Kajiado, reported six-fold increased odds of human seropositivity in households with a seropositive animal compared to those without [12]. Hence, this high incidence was not unexpected given the previously reported 3% and 15% seroprevalence in livestock and humans, respectively, in this county [12]. It is likely some areas in Kenya such as Marsabit with higher *Brucella* sero-prevalence in livestock (13%) and in humans (46%) could have higher incidence in humans and conversely areas with reported low brucellosis sero-prevalence in livestock in former provinces of Western, Nyanza and Nairobi could have lower incidence in humans [11,12,28]. Overall, this high incidence in pastoralist communities suggests significant impacts on socio-economic and livelihoods at household level and on public health systems at community level.

A high proportion of participants presented with arthralgias (63%), back pains (52%), myalgias (20%) and fatigue (37%), all symptoms that might impact the ability to perform the strenuous daily physical activities required for livestock rearing. Further, looking at disease burden in global terms, brucellosis is an acute to chronic debilitating disease but its defined impact metric such as disability weights (a weight factor that reflects the severity of the disease) is not available. However, estimates of disability weights ranging from 0.15 to 0.22 (class II) have been used in a few studies to estimate disability-adjusted life years (DALYs), a measure of overall disease burden associated with human brucellosis [2,29,30]. For example, using brucellosis seropositivity of <2% in India, Singh *et al*. estimated moderate DALYs of 0.29 (95% uncertainty interval 0.08–0.7) per thousand person-years among adults with occupational exposures to livestock [30]. With higher seroprevalence, brucellosis-associated DALYs may be substantially higher in Kenyan pastoralist communities.

We could not determine the brucellosis infection status of 7% (n = 17) of cases who had *Brucella* spp DNA detected but had no detectable antibodies by either IgM, IgG ELISA or RBT assays. Detection of *Brucella* spp DNA has been demonstrated in some patients on brucellosis treatment for up to 6 months after effective treatment, among patients with relapsing brucellosis, and in asymptomatic persons with occupational exposures to *Brucella* in endemic regions [31–33] but these studies did not report the immunologic responses among the patients. None of the enrolled cases in this study reported being on brucellosis treatment. Whereas IgG antibody titers drop with onset of treatment absence of antibody detection among these patients could not be explained. There is the likelihood that some of these 17 sero-negative, *Brucella* PCR-positive individuals are infected with a rough *Brucella* species such as *Brucella canis*, that does not cross react with standard diagnostic agglutination and ELISA tests using capture antigen that only detects antibody from smooth *Brucella* strains [34]. Additionally, though rare, there have been case reports of sero-negative and culture positive or PCR-positive brucellosis cases [23,24,35]. In clinical settings, case management decisions surrounding such diagnostic results are challenging. Given the high proportion (17%) of *Brucella* IgG positives detected in this community, availability of effective diagnostic assays in health centers to inform management would improve timely brucellosis treatment and outcomes. Extensive validation of molecular diagnostic techniques for clinical management in endemic regions would be useful as these assays have increasingly become available in referral facilities.

As expected, malaria was the second most commonly detected pathogen. Other identified etiologies of febrile illness that are unlikely to be diagnosed by clinical presentation alone included West Nile Virus, Rift Valley fever, hepatitis E, *Yersinia pestis* and *Rickettsia spp*. Testing for these pathogens is not routinely conducted in peripheral health facilities. Similar to other studies focusing on etiology of febrile illnesses, the etiology for a large proportion of the patients (68%) was not identified. This was in part because we only collected sera from febrile patients (poorly sensitive for bacterial infections that cause no or low bacteremia) and no other specimens (e.g., stool, urine, nasopharyngeal swabs) that might offer higher sensitivity for detection of some pathogens [18]. Further, only pathogens included in the TAC panel could be detected.

It is important to note the diagnostic and spatio-temporal limitations of extrapolating data reported here. First, bacterial culture which is the confirmatory diagnostic assay for brucellosis was not done. This was due to logistical and biosafety requirements of culturing *Brucella* in the study area. Second, this study was conducted in a geographically limited region and over a one-year period, hence, data obtained may not be extrapolated to national estimates or regions with substantially different risk factors of *Brucella* infection in humans and could vary across years [8]. We also did not adjust the incidence estimated to account for those in the enrolled households who sought medical care elsewhere, or who did not seek care at all despite our biweekly visits. These results however provide insight into the likely high incidence in similar nomadic pastoralist communities living in arid and semi-arid regions in Kenya and the Eastern African region with comparable animal ownership, husbandry and cultural practices.

Globally, brucellosis is a neglected zoonosis, yet not recognized as such [36,37]. In Kenya, brucellosis has been designated a priority zoonotic disease targeted for control [38], but currently a national prevention and control strategy has not been developed and there are no systematic risk reduction activities being implemented. This could in part be due to lack of local disease burden estimates to highlight its public health importance for policy makers. Reducing the burden of human brucellosis can be achieved through strategies aimed at reducing brucellosis in animals mainly though animal vaccination and public health education to adopt risk reduction measures [13,39]. For example, in north-western Greece, five years after introduction of livestock vaccination and public health education, there was large decrease in incidence in humans from 1,100 to 30 cases per 100,000 population by 2002 [26,40]. Data generated here and elsewhere could be useful to advocate for recognition by national and international policy makers and partners in global health, of the need to control the high burden this prevalent endemic zoonosis impacts in rural poor populations in sub-Saharan Africa and other endemic regions.

## Supporting information

S1 Table Distribution of *Brucella* DNA detection using TacMann Array card (TAC) by brucellosis case classification for febrile cases, Kajiado 2015–2016 (n = 236). (DOCX)

S2 Table Demographic, socio, clinical characteristics and history of illness of probable brucellosis cases in Kajiado, Kenya 2015–2016 (n = 39). (DOCX)

S1 Fig Configuration of the TaqMan array card for detection of the agents causing acute febrile illness. (TIF)
