# Supplementary material for: High incidence of human brucellosis in a rural Pastoralist community in Kenya, 2015
Source: PLoS Negl Trop Dis. 2021 Feb 1;15(2):e0009049. doi: 10.1371/journal.pntd.0009049 (PMC7877737; doi:10.1371/journal.pntd.0009049)
Supplement: S1 Table — (DOCX) [file pntd.0009049.s001.docx]

Supplementary Table 1

*Distribution of Brucella DNA detection using* *TacMann Array card (TAC) by brucellosis case classification for febrile cases, Kajiado 2015-2016 (n=236)*

| Case classification | Total tested by TAC  N= 217* | Number with *Brucella spp* DNA detected n(%) |
| --- | --- | --- |
| Confirmed^ᵦ^, n=4 | 2 | 2 (100) |
| Probable^†^, n=39 | 39 | 3 (7.7) |
| Negative, n= 193 | 176 | 17(9.6) |
|  | 217 | 22(10.1) |

^*^19 sera had insufficient volume for TAC testing

^ᵦ^ Confirmed cases tested positive by RBT and IgG *or* RBT and IgM ELISA

**^†^** probable cases tested positive by ELISA (IgG) IgM only *or* IgM only, *but* negative by RBT
