# Supplementary material for: High incidence of human brucellosis in a rural Pastoralist community in Kenya, 2015
Source: PLoS Negl Trop Dis. 2021 Feb 1;15(2):e0009049. doi: 10.1371/journal.pntd.0009049 (PMC7877737; doi:10.1371/journal.pntd.0009049)
Supplement: S2 Table — (DOCX) [file pntd.0009049.s002.docx]

Supplementary Table 2

Demographic, socio, clinical characteristics and history of illness of probable brucellosis cases in Kajiado, Kenya 2015-2016 (n=39)

| Characteristic | n(%) |
| --- | --- |
| Age (median, IQR) Years | 51( 35,64) |
| Gender |  |
| Male | 16(41.0) |
| Female | 23(59.0) |
| Education level completed |  |
| No formal education | 28(71.8) |
| Primary | 7(18.0) |
| Secondary | 4(10.3) |
| College | 0(0) |
| Employment status |  |
| Working on farm | 18(46.1) |
| Non skilled | 12(30.8) |
| Skilled | 5(12.8) |
| Students and minors | 4(10.3) |
| Location of residence |  |
| Arroi | 0(0) |
| Ilmuktani | 13(33.3) |
| Mashuru | 10(25.6) |
| Nkama | 16(41.0) |
| Animal ownership at household level |  |
| Any animal type | 29(74.4) |
| Cattle | 24(61.5) |
| Sheep | 25(64.1) |
| Goats | 29(74.4) |
| Days since onset of symptoms(median, IQR) Years | 7(3,14) |
| Symptoms reported at presentation at health facility |  |
| Fever | 33(84.6) |
| Headache | 31(79.5) |
| Joint pains | 23(59.0) |
| Back pains | 23(59.0) |
| Fatigue | 17(43.6) |
| Abdominal pain | 11(28.2) |
| Muscle pains | 10(25.6) |
| Lack of appetite | 5(12.8) |
| Night sweats | 2(5.1) |
| Similar illness in the last 12 months prior | 18(46.1) |
| How many times have you had a similar illness in the last 12 months |  |
| Once | 6(33.3) |
| 2-3 times | 9(50.0) |
| More than 3 times | 3(16.7) |
| Did you seek care in a health care facility | 17(94.4) |
| Symptoms resolved | 7(38.9) |
