# Supplementary figures and images for: High incidence of human brucellosis in a rural Pastoralist community in Kenya, 2015
Source: PLoS Negl Trop Dis. 2021 Feb 1;15(2):e0009049. doi: 10.1371/journal.pntd.0009049 (PMC7877737; doi:10.1371/journal.pntd.0009049)

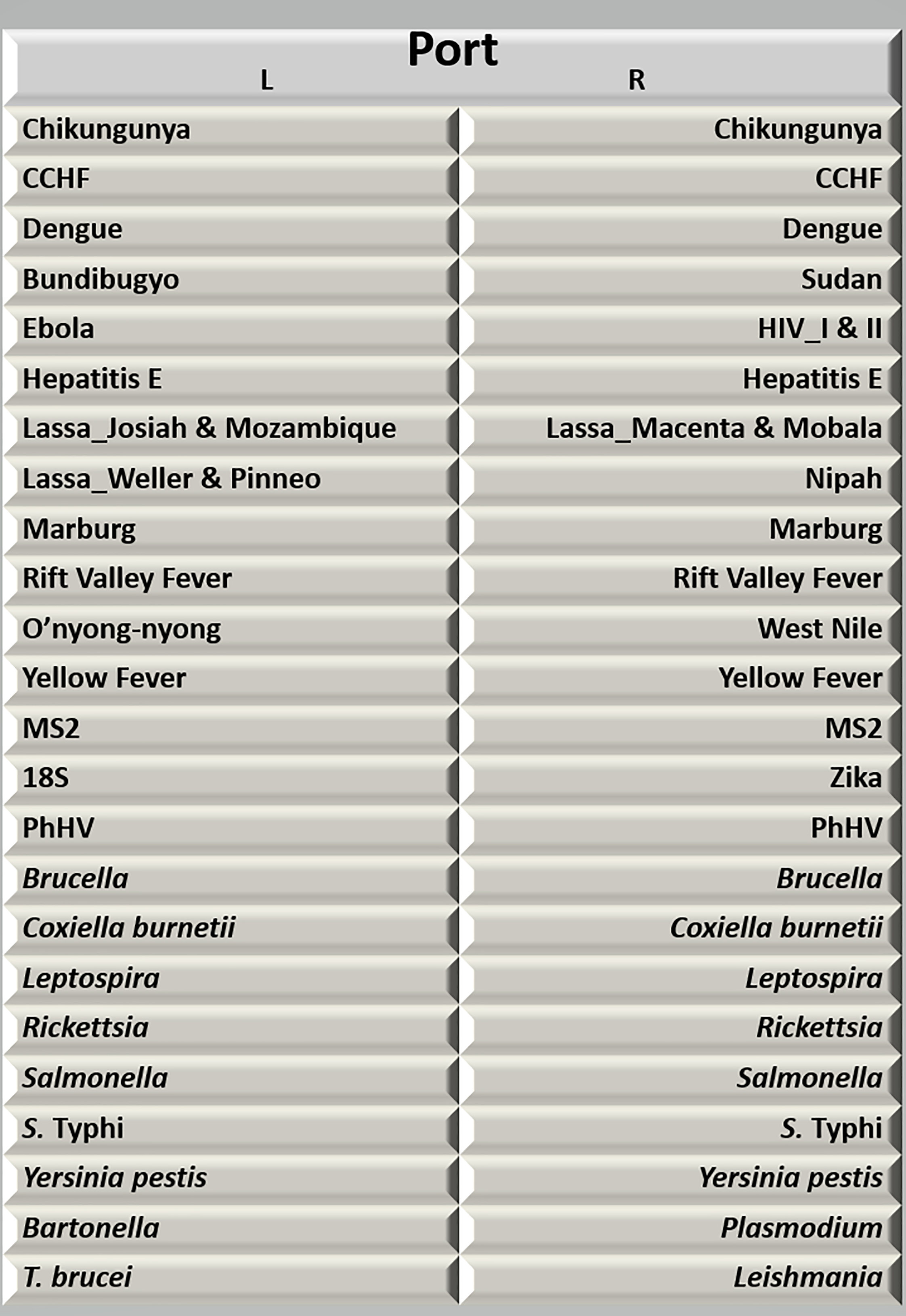

Supplement: S1 Fig — (TIF) [file pntd.0009049.s003.tif]
